# Households across All Income Quintiles, Especially the Poorest, Increased Animal Source Food Expenditures Substantially during Recent Peruvian Economic Growth

**DOI:** 10.1371/journal.pone.0110961

**Published:** 2014-11-05

**Authors:** Debbie L. Humphries, Jere R. Behrman, Benjamin T. Crookston, Kirk A. Dearden, Whitney Schott, Mary E. Penny, on behalf of the Young Lives Determinants and Consequences of Child Growth Project Team

**Affiliations:** 1 Department of Epidemiology of Microbial Disease, Yale School of Public Health, New Haven, Connecticut, United States of America; 2 Department of Economics, University of Pennsylvania, Philadelphia, Pennsylvania, United States of America; 3 Department of Health Science, Brigham Young University, Provo, Utah, United States of America; 4 Department of Global Health, Boston University School of Public Health, Boston, Massachusetts, United States of America; 5 Instituto de Investigación Nutricional, Lima, Peru; Indiana University, United States of America

## Abstract

**Background:**

Relative to plant-based foods, animal source foods (ASFs) are richer in accessible protein, iron, zinc, calcium, vitamin B-12 and other nutrients. Because of their nutritional value, particularly for childhood growth and nutrition, it is important to identify factors influencing ASF consumption, especially for poorer households that generally consume less ASFs.

**Objective:**

To estimate differential responsiveness of ASF consumption to changes in total household expenditures for households with different expenditures in a middle-income country with substantial recent income increases.

**Methods:**

The Peruvian Young Lives household panel (n = 1750) from 2002, 2006 and 2009 was used to characterize patterns of ASF expenditures. Multivariate models with controls for unobserved household fixed effects and common secular trends were used to examine nonlinear relationships between changes in household expenditures and in ASF expenditures.

**Results:**

Households with lower total expenditures dedicated greater percentages of expenditures to food (58.4% vs.17.9% in 2002 and 24.2% vs. 21.5% in 2009 for lowest and highest quintiles respectively) and lower percentages of food expenditures to ASF (22.8% vs. 33.9% in 2002 and 30.3% vs. 37.6% in 2009 for lowest and highest quintiles respectively). Average percentages of overall expenditures spent on food dropped from 47% to 23.2% between 2002 and 2009. Households in the lowest quintiles of expenditures showed greater increases in ASF expenditures relative to total consumption than households in the highest quintiles. Among ASF components, meat and poultry expenditures increased more than proportionately for households in the lowest quintiles, and eggs and fish expenditures increased less than proportionately for all households.

**Conclusions:**

Increases in household expenditures were associated with substantial increases in consumption of ASFs for households, particularly households with lower total expenditures. Increases in ASF expenditures for all but the top quintile of households were proportionately greater than increases in total food expenditures, and proportionately less than overall expenditures.

## Introduction

Over the past two decades, developing countries on average and middle-income countries in particular, have experienced substantial economic growth. As a result, there has been a worldwide convergence in per capita income as developing countries have closed somewhat the gap with high-income countries and the number of people living below international poverty lines has dropped by about a billion [1]. In this global context, a better understanding of the effects of economic growth on consumption of foods that are rich in critical nutrients strengthens understanding of whether policies aimed at improving economic growth lead to improvements in nutritional status.

Relative to plant-based foods, animal source foods (ASFs) are richer sources of accessible protein, iron, zinc, calcium, vitamin B-12 and other nutrients [2]. Their high nutrient density and the bioavailability of the minerals make ASFs particularly important for children in resource-limited settings during critical growth periods. Consuming ASFs is associated with better length- and weight-for-age [3]–[5] and improved cognitive function among children [3], [6]. Studies in Peru have identified micronutrients such as zinc [7], and animal source foods [4] as key influences on improved nutritional status of lower income children. Globally, 25 percent of children under 5 years of age are stunted, and 18 percent are affected by iron deficiency anemia [8]. In Peru, 20 percent of children under 5 years of age are stunted, and 32 percent are anemic [9]. Despite their importance, ASFs on average provide less than 10 percent of total energy intake in most of Sub-Saharan Africa and South Asia and less than 20 percent in most of the rest of the world [10].

Household resources, as reflected in household expenditures as well as income, might be expected to have a major influence on the consumption of ASFs if these foods are highly valued. Because of the importance of ASFs in the diets of children, the question of how responsive the consumption of ASFs is to changes in overall household expenditures is critical for poorer households in the face of income changes. In the economics literature there have been studies of demand systems, where demands for various groups of goods and services that households consume have been shown to depend on household resources (particularly income as reflected in overall expenditures), prices, household demographics, and other factors [11]–[15]. Ernst Engel (1821–1896) was the first to systematically investigate the relationship between goods expenditure and income, and in 1857 proposed “Engel's Law” stating that the poorer a family is, the larger the budget share it spends on food. A corollary of that law is that as income increases, expenditures on nourishment (food) increase by smaller percentages than the percentage increase in income. The demand for such commodities is said to be inelastic (see discussion of “elasticities” below). In addition, as incomes rise “Bennett’s Law” states that food expenditures will favor more nutrient-rich foods, such as animal source foods, and food expenditures on starchy staples will decrease [16]. However, as Abdulai and Aubert [17] note in sub-Saharan Africa, evidence on responsiveness to expenditures for individual food and food groups in developing countries is limited. Most of the existing evidence, such as Bhaumik and Nugent’s analysis of competing demands for food and livestock feed in Peru [18] and the recent analysis of price influences on demand for animal products in the BRIIC countries (Brazil, Russia, India, Indonesia, China) [19], are based primarily on cross-sectional associations rather than longitudinal analysis. Estimates resulting from cross-sectional analyses may confound expenditure effects with unobserved household characteristics such as preferences for different types of food. To our knowledge, moreover, there is no published evidence based on longitudinal analyses with control for unobserved household fixed factors during periods of fairly rapid overall economic growth in developing countries.

There are many studies consistent with Engel’s Law for total food consumption in different time periods and countries [11]–[15]. But food is not homogenous. At very low incomes households may consume largely basic staples. With more income they may increase the shares of other foods, such as green leafy vegetables, fruits and animal source foods [20], [21]. When demand studies disaggregate food into food groups, they have found variations in income elasticities, with some foods (such as basic staples) responding relatively little to income changes (income inelastic) and others responding proportionally more than income changes (income elastic) [17], [21].

Our basic methodological question in this study is whether household fixed-effects estimates are preferred to random-effects estimates. If they are, the cross-sectional estimates that predominate in the previous literature may be confounded if, for example, patterns in unobserved preferences for different types of food are associated with household expenditures.

We analyzed: (1) how ASF expenditures change with changes in household resources in a middle-income country (Peru) during a period of substantial economic growth? and (2) to what extent are these changes dependent on whether a household is poor or relatively better off? If increases in household resources lead to substantial increases in ASF expenditures for low-income households, dietary quality and perhaps quantity improves, with the potential for decreasing malnutrition. Peru experienced rapid overall income growth during the period of the study (2002–2009), with a 40 percent increase in Peruvian Gross National Product per capita measured in constant 2005 international purchasing power parity (PPP) terms and a decrease from 24.2 percent to 14.0 percent of the prevalence of poverty (percentage of the population living below $2 per day per capita in PPP terms) [22]. Improvements between 2002 and 2009 are also apparent among Young Lives (YL) households, as 83.7 percent reported increased total expenditures per adult equivalent (AE) in 2009. Peru has also seen a dramatic decrease in stunting rates, from 29.8 percent in 2005 to 18.1 percent in 2011 [23].

The term “elasticity” is used to describe responsiveness of one variable X to another variable Y. The elasticity of X in response to Y is the percentage change in X given a specific percentage change in Y. For example, the elasticity of ASF expenditures with respect to food expenditures is the percentage change in ASF expenditures for a given percentage change in food expenditures. If this elasticity is 0.80, then ASF expenditures increase by 8 percent for a 10 percent increase in food expenditures. If the elasticity is 1.0, the percentage change in ASF equals the percentage change in food expenditures. A food demand with an elasticity less than 1.0 is called “inelastic” while a food demand with an elasticity greater than 1.0 is called “elastic”. Consumer demands for basic commodities such as food staples are generally inelastic and they are likely to account for a considerable share of consumption expenditures at low overall expenditure or income levels, but increase less than proportionately as overall expenditure or income increases. However the demand elasticities for some food items, perhaps including foods rich in micronutrients such as ASF, are likely to be higher than for basic staples and for food in general. If household resources are very low, food consumption tends to concentrate on cheap sources of basic macronutrients that are necessary for survival. But with more household resources, people choose to diversify their food consumption because of their implications for health beyond survival and/or because they prefer diversity in their diet. [21], [24].

## Methods

### Ethics statement

The original protocol and each subsequent round of YL data collection was approved by the Ethics committees of the Instituto de Investigación Nutricional and the University of Oxford. Ethical approval for this analysis was obtained from the University of Pennsylvania Institutional Review Board.

### Study design and participants

The Peruvian Young Lives Younger Cohort (YL-YC) study is a part of the YL 4-country study following children and their households. The YL study so far consists of three waves of data collection in 2002, 2006, and 2009, hereafter referred to as rounds 1–3, respectively. Households in the Peruvian cohort were enrolled in 20 districts randomly selected from all but the 5 percent of wealthiest districts in Peru [25]. Details of the YL study have been described [26] and can be found at http://www.younglives.org.uk. For each round, trained enumerators visited each household and completed detailed surveys of household expenditures. The prices and availabilities of a standard list of items from shops in each community were collected. We utilized YL-YC data from all Peruvian households that had complete household expenditure data in all three years (n = 1750). Peru was the only YL country with food expenditure data for all three rounds.

### Study indicators

#### Expenditures

Total expenditures, food expenditures, and ASF expenditures were calculated for all three rounds. Each household was asked about total expenditures and categories of expenditures over the previous two weeks. Detailed information was collected on food expenditures, covering 33 different food groups. Animal source food groups included: red meat and processed meat, poultry, fish, milk, yogurt, cheese, and eggs. Three of the eight categories of ASF (milk, yogurt, cheese) were combined into one group (dairy) because of low levels of consumption of yogurt and cheese, and processed meat was combined with red meat. Locality- and goods-specific consumer price indices from Peru’s Instituto Nacional de Estadística e Informática (INEI) were used to adjust the 2002 and 2009 household food expenditures to 2006 food prices in order to control for location- and goods-specific price changes. Total expenditures were deflated to 2006 levels. Household adult equivalents (AE) were calculated [27], and all expenditures were expressed per AE.

### Statistical analyses

All data were analyzed using SPSS for Windows (version 19.0, 2011, IBM) and Stata (version 12.0, 2011. Stata Corp).

We compared the cross-sectional relations between overall food expenditures, ASF expenditures, and household consumption at different levels of household expenditures for 2002, 2006 and 2009 using ANOVA. Differences between years were tested with a paired t-test or paired Wilcoxon rank sum test, adjusted for multiple comparisons.

We estimated multivariate relations for each specification among overall food expenditures, ASF expenditures, and household resources using two sets of data: 2002 and 2006, and 2006 and 2009. Our preferred estimates control for unobserved household fixed effects, but to test whether those estimates are preferred with our data and to investigate possible associations with other observed controls such as maternal schooling we also undertook household random effects, with and without observed controls.

### Model framework

**Figure 1:**
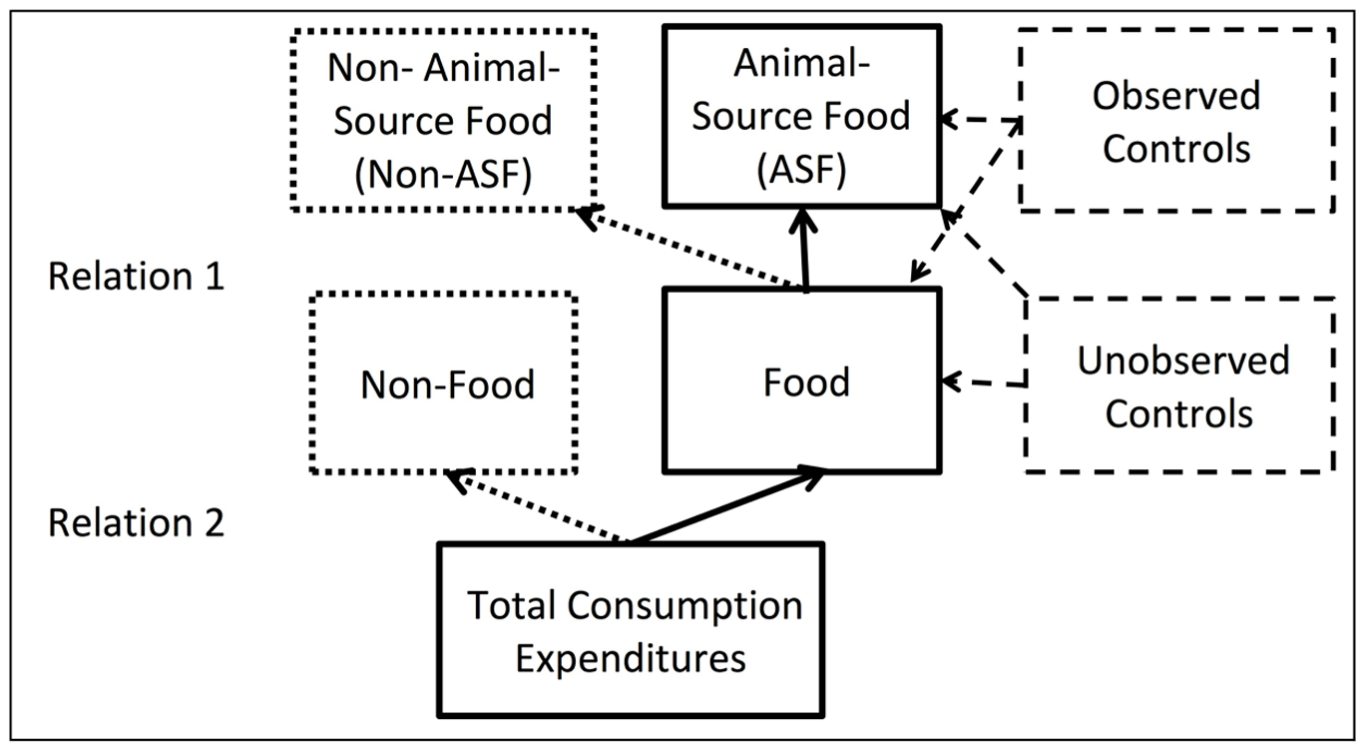
Framework for Analysis. Relation 1 represents the division of food expenditures between ASF and non-ASF, as shown in the top row. Relation 2 represents the division of total expenditures between food and non-food expenditure, as shown in the middle row. We focused our attention on the solid arrows between the center boxes in the figure. Observed controls (dashed boxes on the right) included other important determinants of ASF and food consumption expenditures, such as maternal schooling [31], [49], paternal schooling and community wealth. Unobserved controls included other household characteristics, such as food preferences.

We used total household consumption expenditures to represent long-term household resources. This was done as income tends to fluctuate from year to year, particularly in poor households, but households can use savings and withdrawals from savings to smooth resources so that total household consumption expenditures better represent longer-run household resources than does annual income [28], [29]. We represented the relations between ASF expenditure and total household consumption expenditures as expressed in the conceptual framework presented in Figure 1. Each of the two relations pertains to a division between two categories of expenditures:

Relation 1: division of food expenditures between ASF and non-ASF.
Relation 2: division of total expenditures between food and non-food expenditures.

We estimated these two relations as they pertain to the center, solid boxes in Figure 1. Further details and equations are available in Appendix S1. We were interested in questions such as, if food expenditures increase by 10 percent, what happens to ASF expenditures? And, does it matter if the households have relatively low or relatively high overall expenditures? Because of the dichotomous division of food expenditures into ASF and non-ASF food expenditures, if we learned that ASF expenditures increase by, for example, more than 10 percent when total food expenditures increase by 10 percent, then necessarily non-ASF food expenditures must increase by less than 10 percent. Although we focused our attention on the solid arrows between the center boxes in the figure, we also learned about the dotted arrows pointing to the dotted (left) boxes in the figure. Observed controls included other important determinants of ASF and food consumption expenditures, such as maternal schooling [30], [31], paternal schooling and community wealth. Unobserved controls included all other household characteristics, such as food preferences. We were interested in these controls for two reasons. First, some controls, such as women’s schooling, have previously been reported to impact household food purchases, so we undertook random effects estimates with and without them to see if their exclusion confounds the estimates of interest [30], [31]. Second, we wanted to avoid confounding the estimates of the elasticities for the primary variables of interest as happens in cross-sectional estimates when correlated controls are excluded. Indeed an important contribution of our study is to avoid such confounding with factors such as unobserved preferences that may be correlated with income and expenditures.

We also estimated parallel relations for each of the five types of ASF. Expenditures on each type of ASF were the dependent variables and total ASF expenditures and total expenditures were the key right-side variables. Elasticities for individual ASF component expenditures were constrained to be consistent with the elasticity for total ASF expenditures, using the identity that the elasticity for total ASF expenditures is a weighted-average of the elasticities of the ASF component expenditures with the shares of the components in the total ASF expenditures as the weights.

### Multivariate specifications

In the economics literature on commodity demands, two of the most commonly-used functional forms are linear and log-linear. The former assumes constant marginal consumption propensities (and varying elasticities) and the latter assumes constant elasticities. Both can be viewed as first-order Taylor series approximations to more general specifications. We used natural logarithm (ln) functional forms to estimate elasticities because of the straightforward relation between ln functional forms and elasticities. In the ln-linear case, the coefficient estimate of the predictor gives a direct estimate of the elasticity of the dependent variable with respect to that predictor. Since the ln-linear form constrains the elasticity to be the same for all values of the predictor, we added the square of the ln predictor to permit nonlinear elasticities.

We began by estimating the sequence of two relations pertaining to the solid arrows between the center boxes in Figure 1 discussed above, (1) ASF expenditures as a function of food expenditures, and (2) food expenditures as a function of total expenditures (see Appendix S1 for details). While estimates of relations (1) and (2) are of interest to better understand the sequence through which total consumption expenditures may affect ASF expenditures through food expenditures, it also is of interest to know the direct elasticity of ASF expenditures with respect to total consumption expenditures, which can be obtained from estimating (3) ASF expenditures as a function of total expenditures. For each of the three relations we estimated three models: (a) random effects, (b) random effects with observed controls, and (c) fixed effects. Household fixed-effects estimates control for all fixed characteristics of the household, whether observed or not. If there are important unobserved fixed characteristics, such as the fixed component of preferences, these fixed-effect estimates avoid confounding that might occur in the absence of such controls. Household fixed effects also control for any secular trends in time-varying unobservables that are common across observations – but not for time-varying unobservables that differ systematically across households such as ones that affected poorer households differently from better-off households. We report Hausman’s p that tests whether the fixed-effects models are preferred over the random-effects models with controls. Based on the estimates of the multivariate relations, we next estimated elasticities of ASF expenditures under multiple scenarios as detailed below.

In preliminary estimates we analyzed the robustness of our estimates to some alternatives: for instance, distinguishing between rural and urban areas, allowing the elasticities to vary by the tertiles of the right-side variables rather than including the squares of those variables, and allowing the elasticities to vary by the tertiles of the wealth asset index. We were not able to identify time-varying changes in elasticities between the different rounds. There were no significant differences between the rural and urban coefficients, and the tertile estimates were consistent with the estimates with linear and squared ln terms.

## Results

### Changes in Food and ASF Expenditures between 2002 and 2009

Table 1 provides descriptive statistics for 2002, 2006 and 2009. Between 2002 and 2009, total expenditures per adult equivalent increased by 109 Peruvian soles or USD 31.24 (in constant 2006 prices, September 2006 exchange rate), and total food expenditures per AE increased by 14.1 soles (USD 4.04). Food expenditures as a percentage of total expenditures decreased dramatically between 2002 and 2009 (47% to 23.2%). ASF expenditures increased by 8.6 soles (USD 2.46) from 2002 to 2009. Changes between 2002 and 2009 were significant for all food components except the proportion of the food budget devoted to meat and the proportion of the food budget used for dairy. Dairy, poultry, and meat together accounted for most of the ASF expenditures.

**Table 1:** Average Household Expenditures on Food and Groups of ASF per Adult Equivalent (AE).

	2002	2006	2009
	Mean 2006 soles (SD) or Mean % (SD)*		
Total 15-day expenditures (2006 soles) per AE	326.2 (548.2)^a^	356.0 (238.7)^a^	453.5 (240.2)^b^
Total 15-day food expenditure per AE	82.5 (65.3)^a^	85.6 (47.1)^a^	96.6 (43.2)^b^
Food expenditure as % of total expenditure	46.6% (21.0)^a^	25.9% (6.1)^b^	23.0% (6.4)^c^
ASF 15-day expenditure per AE	26.0 (24.5)^a^	26.1 (19.4)^a^	34.6 (21.3)^b^
ASF expenditures as % of total food expenditures	29.7% (13.6)^a^	28.9% (12.1)^b^	34.1% (11.3)^c^
15-day meat expenditure per AE	7.0 (16.9)^a^	6.4 (8.6)^a^	9.1 (10.0)^b^
15-day meat % of ASF	23.2% (23.2)^a^	21.5% (21.2)^b^	23.9% (19.9)^a^
15-day egg expenditure per AE	2.2 (2.1)^a^	2.2 (2.0)^a^	2.57 (2.1)^b^
15-day egg % of ASF	12.7% (17.2)^a^	11.4% (12.9)^a^	9.3% (9.9)^b^
15-day dairy expenditure per AE	8.2 (9.0)^a^	8.3 (7.9)^a^	10.4 (8.5)^b^
15-day dairy % of ASF	30.8% (23.4)^a^	32.3% (21.6)^b^	30.3% (17.4)^a^
15-day poultry expenditure per AE	6.3 (7.2)^a^	5.8 (6.2)^a^	8.60 (7.8)^b^
15-day poultry % of ASF	24.3% (21.6)^a^	21.8% (18.5)^b^	24.7% (17.1)^c^
15-day fish expenditure per AE	2.3 (3.7)^a^	3.3 (4.5)^b^	3.9 (4.3)^c^
15-day fish % of ASF	9.0% (13.3)^a^	13.0% (14.1)^b^	11.9% (11.4)^b^

*2002, 2006 and 2009 columns represent the mean in soles or mean percent of expenditures (SD).

a,b,c Columns with a different letter are significantly different (p<0.05) in a matched comparison of means. Mean expenditures were compared with a pairwise t-test, and percentages were compared with the Wilcoxin signed rank test for non-parametric data.

**Figure 2:**
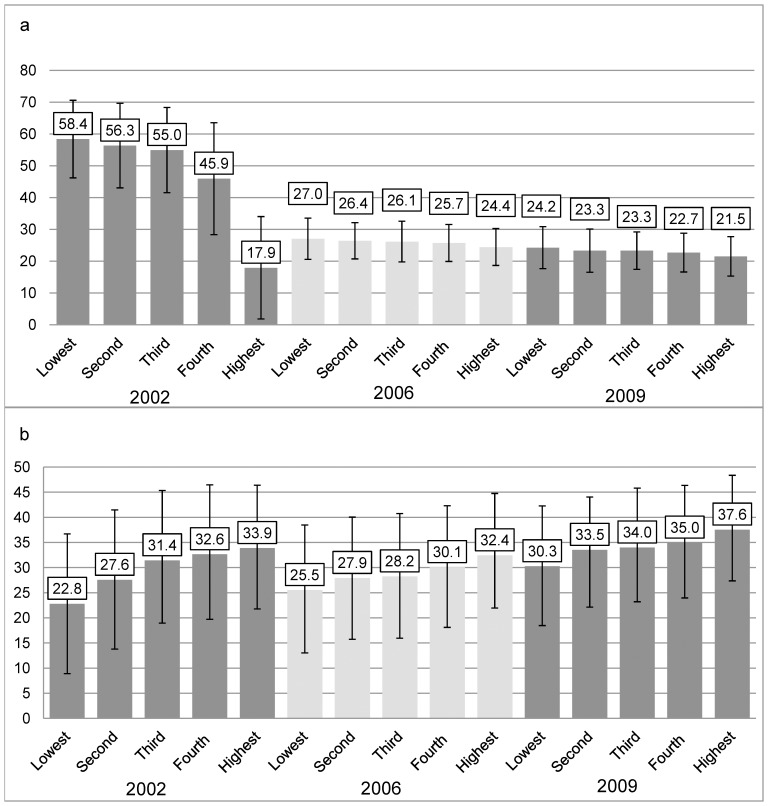
(a) Percent total expenditures devoted to food in 2002, 2006 and 2009, and (b) percent food expenditures devoted to ASF in 2002, 2006 and 2009, by 2002 quintiles of total expenditures. (**a**) In 2002 each quintile is significantly different from all other quintiles in that year (p<0.05) except for the first and second quintiles, and the second and third. In 2006 each quintile is significantly different from most other quintiles in that year (p<0.05). In 2009 the lowest quintile is significantly different from all other quintiles, and the second, third and fourth quintiles are significantly different from the highest quintile (p<0.05). All quintiles are based on 2002 total expenditures. (**b**) In 2002 each quintile is significantly different from all other quintiles in that year except for the third and fourth quintiles, and the fourth and highest quintiles (p<0.05). In 2006 each quintile is significantly different from all other quintiles for that year except for the second and third quintiles. In 2009 most quintiles are significantly different from all other quintiles in that year (p<0.05). All quintiles are based on 2002 total expenditures.

Households in the lower quintiles of total expenditures spent a higher percentage on food (Figure 2a). In 2002, these proportions for all quintiles were different (p<0.05) except for the lowest and second quintiles, and the second and third quintiles. In 2009 all quintiles were significantly different from the highest, and the lowest quintile was also significantly different from the fourth quintile (p<0.05). Households from the lower quintiles of total expenditures devoted smaller percentages of their overall food expenditures to ASFs (Figure 2b). In 2002 all quintiles were significantly different except for the third and fourth quintiles and the fourth and highest quintiles. In 2009 all were significantly different except the second and third, second and fourth, and the third and fourth quintiles (p<0.05).

Figure S1a–c shows the percentage of ASF expenditures devoted to each type of ASF. Spending was significantly different (p<0.05) across quintiles for poultry, meat and eggs in 2002, and for eggs in 2009.

### Elasticities for Total ASF Expenditures

Table 2 gives the basic estimates for the three relations estimated (relations (1)–(3) in Appendix S1), in each case with random effects and no controls in the first column (Model 1), random effects with controls in the second column (Model 2) and fixed effects in the third column (Model 3). In addition to the patterns in elasticities that are of primary interest, two basic features of these estimates emerge. First, the estimates for the random effects with controls are very similar to the estimates of random effects without controls. Therefore, in terms of the estimates of the elasticities of interest, parental schooling and community wealth do not add much to the explanatory power of the relation nor change much the estimated coefficients of the right-side food expenditure and total consumption expenditure variables of primary interest. Second, in each case, the fixed-effects estimates are preferred over the random-effects estimates at the <0.01 level based on the Hausman test comparing fixed-effects and random-effects models.

**Table 2:** Multivariate analysis of ASF expenditures with total and food expenditures as predictors.

Dependent Variable		Predictor Variable	Random Effects	Random Effects with Controls	Fixed Effects	Elasticity at Given Percentiles				
2002–2006						10%	25%	50%	75%	90%
1	ASF	Food	3.04	3.06	3.13					
		(Food)^2^	−0.20	−0.21	−0.23					
		R^2^	0.64	0.65	0.64					
		Hausman p			<0.001	1.48	1.32	1.15	1.00*	0.85
2	Food	Consumption	1.64	1.66	1.16					
		(Consumption)^2^	−0.11	−0.11	−0.08					
		R^2^	0.49	0.52	0.49					
		Hausman p			<0.001	0.45	0.38	0.29	0.21	0.12
3	ASF	Consumption	2.20	2.28	1.48					
		(Consumption)^2^	−0.15	−0.16	−0.11					
		R^2^	0.32	0.38	0.32					
		Hausman p			<0.001	0.50	0.41	0.29	0.17	0.06
**2006–2009**						**10%**	**25%**	**50%**	**75%**	**90%**
1	ASF	Food	2.69	2.75	2.64					
		(Food)^2^	−0.16	−0.17	−0.17					
		R^2^	0.67	0.69	0.67					
		Hausman p			<0.001	1.37	1.25	1.13	1.03	0.94
2	Food	Consumption	2.33	2.32	2.17					
		(Consumption)^2^	−0.14	−0.14	−0.13					
		R^2^	0.72	0.72	0.72					
		Hausman p			<0.01	0.84	0.75	0.65	0.55	0.46
3	ASF	Consumption	3.89	3.93	3.52					
		(Consumption)^2^	−0.24	−0.25	−0.22					
		R^2^	0.56	0.57	0.56					
		Hausman p			<0.001	1.27	1.11	0.95	0.78	0.54

All elasticities are significantly different from 0 (p<0.001); *Elasticity is not significantly different from 1 (p>0.01).

**Figure 3:**
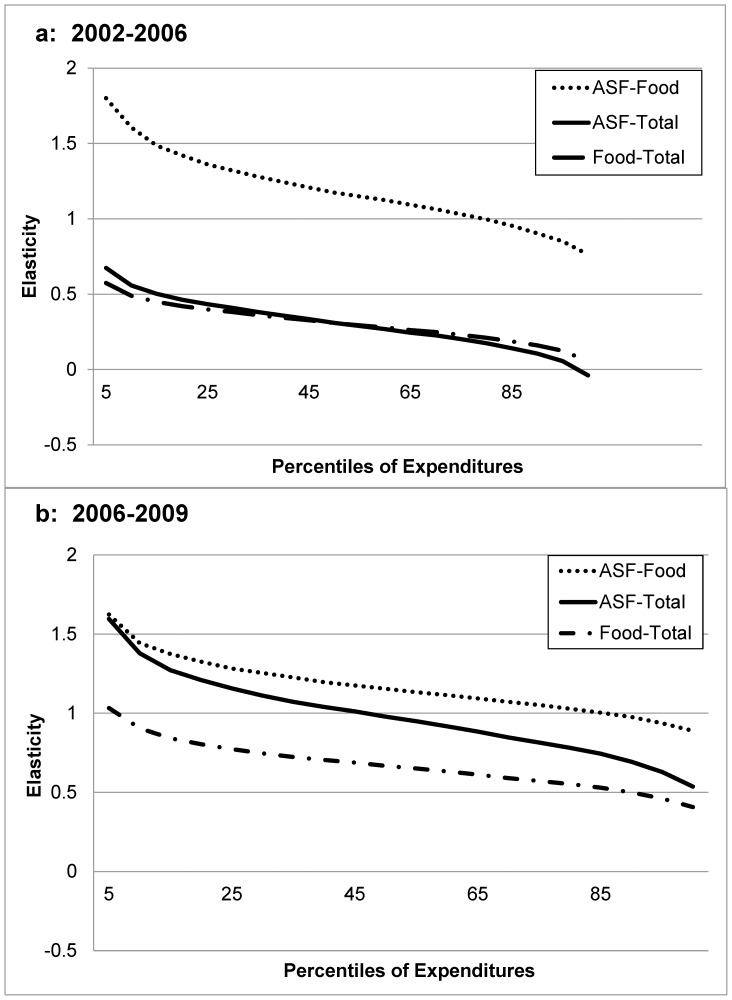
Elasticities of ASF and Food Expenditures with respect to Food or Total Expenditures. (**a**) From 2002–2006 elasticities are significantly different from 0 (p<0.05) except for ASF-Total and Food-Total >95^th^ percentile. All are significantly different from 1.0 (p<0.05) except for ASF-Food between percentiles 65 and 80. (**b**) From 2006–2009 all elasticities are significantly different from 0 (p<0.05). All are significantly different from 1.0 (p<0.05) except for ASF-Food between percentiles 75 and 98, Food-Total below the 5^th^ percentile, and ASF-Total between percentiles 35 and 45.

The preferred fixed-effects estimates (as well as the random-effects alternatives) suggest for all three relations higher elasticities for poorer households (as reflected in the larger coefficient for the ln linear term) and larger declines in the elasticities as expenditures increase (as reflected in the absolute magnitudes of the coefficients for the squared ln terms). The right-side of Table 2 gives the elasticities for the three fixed-effects relations at the 10th, 25th, 50th, 75th and 90th percentiles, respectively, of the distributions for the right-side expenditure variables (food expenditures for relation 1; total expenditures for relations 2 and 3). Figure 3 graphs the three sets of elasticities against the percentiles of the relevant expenditures.

The elasticities of ASF expenditures with respect to food expenditures (relation 1) are similar in the two time periods: above 1 at lower percentiles of food expenditures, and not significantly different from 1 at the higher income percentiles. Thus those at the low end of the food expenditure distribution increase their expenditures on ASF more than the increase in food expenditures overall, and those high in the distribution change their ASF expenditures with the same percentage as their food expenditures. In 2002–2006 the elasticities of food expenditures with respect to total consumption expenditures are always significantly different from 1.0, and in 2006–2009 the elasticities of food expenditures with respect to total consumption expenditures are significantly different from 1.0 at all expenditure percentiles except below the 5^th^ percentile. Relation (3) combines the response of ASF expenditures to food expenditures in relation (1) and the response of food expenditures to overall consumption expenditures in relation (2) to give the elasticities of ASF expenditures with respect to overall consumption expenditures. These elasticities are below 1 across all percentiles of expenditures in 2002–2006, and in 2006–2009 are above 1 for the lower expenditure percentiles, not significantly different from 1 between the 35^th^ and 45^th^ percentiles, and less than 1 above the 45^th^ percentile.

Therefore, between 2002 and 2006 ASF expenditures increase 5.0 percent at the 10th percentile in response to a 10 percent increase in total consumption expenditures, due primarily to a relatively large increase in ASF expenditures with respect to food expenditures and a smaller proportional increase in total food expenditures in response to total consumption expenditure. In contrast, at the 90^th^ percentile, the increase in ASF expenditure is only 0.6 percent with a 10 percent increase in total consumption expenditure, due to a relatively small increase in ASF expenditure relative to total food expenditure and a relatively smaller increase in total food expenditure relative to overall consumption expenditure. Similar patterns are observed between 2006 and 2009.

Despite the limited explanatory power of the observed controls and the fact that their inclusion does not change the expenditure elasticities of primary interest, the effects of these controls are of interest in themselves. At levels of maternal schooling above primary school, more schooling is associated with a greater proportional increase in ASF expenditures than food expenditures, and total consumption expenditures (Table 3). At levels of paternal schooling above primary school in 2002–2006, and secondary school in 2006–2009, more schooling is associated with a greater proportional increase in ASF expenditures with reference to food expenditures and increases in both ASF expenditures and food expenditures with reference to total consumption expenditures. The effect is slightly smaller for paternal schooling than it is for maternal schooling. Community wealth in 2002–2006 is associated with a greater proportional increase in ASF expenditures with reference to total expenditures than ASF expenditures with reference to food expenditures and total expenditures.

**Table 3:** Controls for Random Effects Models.

	ASF-Food	Food-Total	ASF-Total
	2002–2006 Estimates		
Community Wealth	0.015*	0.016**	0.035**
Maternal Schooling			
6 grades	0.064*	0.010	0.086*
7–12 grades	0.158**	0.087**	0.254**
>12 grades	0.213*	0.180**	0.389**
Paternal Schooling			
6 grades	0.012	0.040	0.058
7–12 grades	0.068*	0.064*	0.135*
>12 grades	0.146*	0.165**	0.324**
	**2006–2009 Estimates**		
Community Wealth	0.012*	0.001	0.010*
Maternal Schooling			
6 grades	0.05	0.004	0.06
7–12 grades	0.139**	−0.019	0.113**
>12 grades	0.179*	−0.030	0.122
Paternal Schooling			
6 grades	−0.042	0.023	−0.012
7–12 grades	0.051	0.011	0.064*
>12 grades	0.116*	−0.002	0.089

*p<0.05; **p<0.001.

### Elasticities for Specific ASF Foods

**Figure 4:**
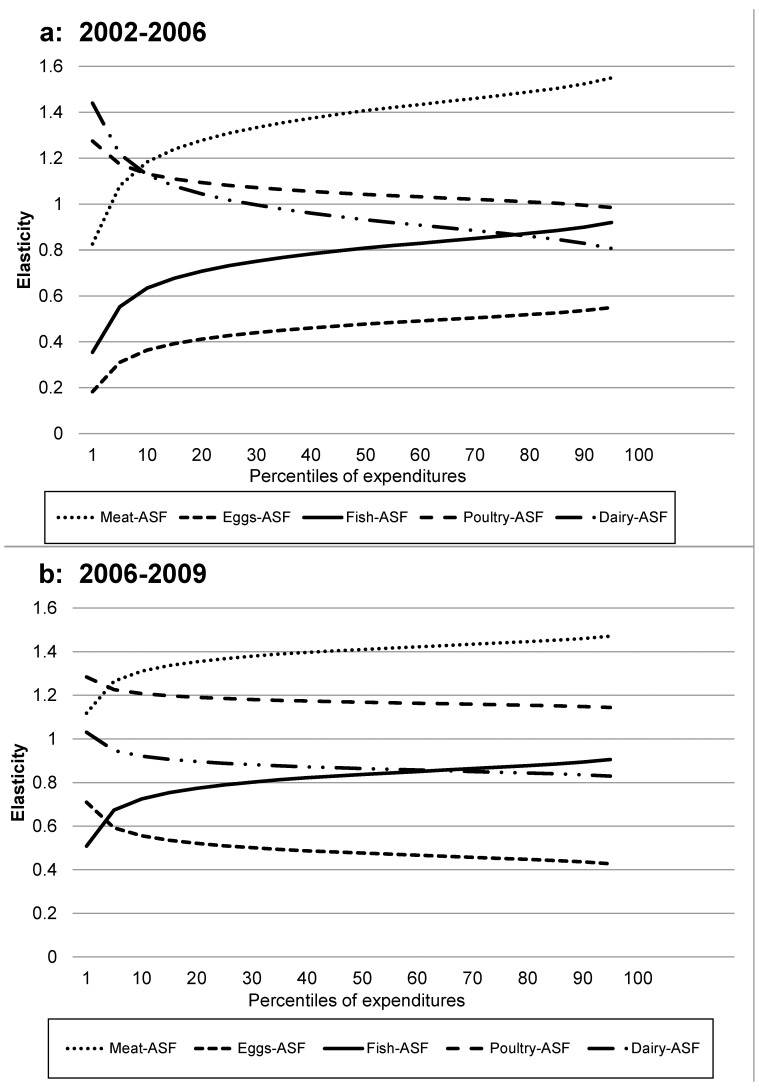
Elasticities of ASF components with reference to total ASF expenditures. Elasticities for individual ASF component expenditures are constrained to be consistent with elasticity for total ASF expenditures. (**a**) From 2002–2006 elasticities are not significantly different from 1 for meat below the 10^th^ percentile, for poultry above the 15^th^ percentile, for dairy between the 15^th^ and 75^th^ percentiles, and for fish >70^th^ percentile. Elasticities with respect to total ASF expenditures are always significantly different from 1 for eggs. (**b**) From 2006–2009 elasticities are not significantly different from 1 for fish between the 10^th^ and 25^th^ percentiles. Elasticities with respect to total ASF expenditures are always significantly different from 1 for meat, poultry, dairy and eggs.

Table 4 and Figure 4 give the elasticities for the types of ASF expenditures with respect to total ASF expenditures. In both 2002–2006 and 2006–2009, meat and fish expenditures have increasing elasticities at higher total ASF expenditures, while poultry and dairy expenditure have decreasing elasticities at higher total ASF expenditures. Eggs show a decrease across the percentiles of total ASF expenditures in 2002–2006 and an increase across the percentiles in 2006–2009. In 2002–2006 the elasticities for the components of ASF expenditures with respect to total ASF expenditures are not significantly different from 1 for meat below the 10^th^ percentile, poultry above the 10^th^ percentile, dairy between the 15^th^ and the 75^th^ percentiles, and for fish above the 70^th^ percentile. In 2006–2009 elasticities are significantly different from 1 for all components except fish at and above the 90^th^ percentile. Aside from these exceptions, the shares of the ASF component expenditures in total ASF expenditures change differentially over the distribution of the total ASF expenditures. Expenditures on eggs and fish over the entire range of total ASF expenditures in both time periods, and dairy in 2006–2009, increase proportionately less than total ASF expenditures as total ASF expenditures increase. Expenditures on meat in both time periods increase by larger percentages as total ASF expenditures increase.

**Table 4:** Elasticities of ASF components with reference to ASF expenditures and total expenditures at specified percentiles of total expenditures.^a^

Percentile	Meat	Poultry	Dairy	Eggs	Fish
2002–2006					
ASF component-Total ASF Expenditures					
10	1.184	1.131	1.127	0.363	0.634
25	1.310	1.081**	1.017**	0.427	0.732
50	1.407	1.042**	0.931**	0.477	0.809
75	1.474	1.015**	0.873**	0.511	0.861**
90	1.524	0.995**	0.829	0.536	0.900**
**ASF component-Total Consumption**					
10	0.828	0.658	0.781	0.444	1.095**
25	0.684	0.551	0.625	0.364	0.890**
50	0.499	0.413	0.425	0.262	0.627
75	0.323	0.281	0.235	0.165	0.376
90	0.139***	0.144***	0.035***	0.063***	0.115***
**2006–2009**					
**ASF component-Total ASF Expenditures**					
10	1.311	1.208	0.921	0.555	0.725
25	1.368	1.185	0.888	0.510	0.789
50	1.410	1.168	0.864	0.476	0.837
75	1.440	1.157	0.847	0.452	0.870
90	1.460	1.148	0.835	0.436	0.893**
**ASF component-Total Consumption**					
10	1.524	1.555	1.226	0.855**	1.097**
25	1.369	1.342	1.119**	0.738	0.966**
50	1.211	1.125**	1.010**	0.620	0.832
75	1.050**	0.903**	0.899**	0.499	0.695
90	0.902**	0.699	0.230**	0.388	0.569

a Elasticities for individual ASF component expenditures are constrained to be consistent with elasticity for total ASF expenditures.

**Elasticity is not different from 1, p value>0.05.

***Elasticity is not different from 0, p value>0.05.

While the overall patterns are similar in the two time periods, there are some interesting differences. In 2002–2006 dairy and poultry elasticities with respect to total ASF expenditures are not significantly different from 1 across much of the expenditure percentiles, in contrast to 2006–2009 when the elasticities are significantly above 1 for poultry and significantly below 1 for dairy. In addition, eggs showed an increasing elasticity with ASF expenditure percentiles in 2002–2006, and a decreasing elasticity with ASF expenditures in 2006–2009. Dairy elasticities decreased with ASF expenditures in both time periods, and the elasticities were lower across lower ASF expenditure percentiles in 2006–2009.

## Discussion

Based on our preferred household fixed-effects estimates in a fairly rapidly growing economy, we found that 1) relative to households in the highest quintile of expenditures, lowest quintile households dedicated a greater percentage of overall spending to food; 2) households from the lowest expenditure quintiles devoted a smaller percentage of their overall food expenditures to ASFs; 3) households in the lower quintiles of expenditures showed a greater increase in ASF expenditures with an increase in food expenditures (elasticity >1); and 4) households in the higher quintiles of consumption showed a less than proportional increase in ASF expenditures (elasticity <1) with increases in total consumption expenditures.

Similarly, Abdulai and Aubert [17] found that in Tanzania the diets of high-income households were richer in all micro- and macronutrients. Though they did not specify whether micro- and macronutrients were from animal sources, they did report that the commodity groups most responsive to expenditure fluctuations were animal source foods including meat, fish and eggs, and milk products, and that elasticities were lower for those micronutrients that are consumed through staple foods and higher for micronutrients obtained mainly through animal products. Likewise, Ecker and Qaim [32] generally found that in Malawi, higher household incomes were associated with a more diversified diet as measured by the number of different food items consumed.

Our findings from Peru indicate that increases in total expenditures are associated with greater increases in consumption of ASFs for households in the lowest quintiles of expenditures, a finding also reported by Ecker and Qaim [32] in urban Malawi. In addition to increasing substantially the demand for ASFs, increases in household resources for poorer households allow such families to purchase greater quantities of or higher quality healthcare, education, water and sanitation, and so on [32]–[34]. However, several studies [33], [35] show that growth in income is more effective in improving nutritional status when coupled with nutritional education than when there is an increase in income alone.

Increases in ASF consumption have been shown to contribute to improved performance on school tests [36], improved cognitive functioning for undernourished children [3], and improved anthropometric indices [3]. However, a recent intervention that provided 6–18 month old children with 30–45g of meat daily for twelve months did not find a treatment effect on linear growth [37]. The authors suggest the lack of effect may be due to the high rates of undernutrition in the population (mean −1.4 LAZ at 6 months) [37]. This recent finding highlights the challenge of determining what levels of dietary ASF are needed to have public health impact, and in interpreting the implications of changes in ASF spending.

There is some concern that the health and nutritional benefits of increasing ASF consumption might have negative environmental costs due to the resources needed for animal production as well as animal waste and other negative environmental impacts [38]. Other reports have suggested that this is a very complex question, with potential changes in natural ecosystems, zoonoses associated with livestock farming, as well as climate and greenhouse gas emissions [36]. A recent analysis highlights the heterogeneity of livestock production systems, with eight different ruminant systems identified, and a range in environmental impacts across the systems [39]. Hence, the impact of increasing consumption of animal source foods on environment depends on the livestock production systems in use.

We found that household fixed-effects estimates of demand for ASF are preferable to random-effects estimates. This means that there are unobserved factors such as preferences that significantly affect household demands for ASF and for total food that may confound the cross-sectional estimates that dominate in most of the literature.

This study had several limitations. The YL-YC study data is limited to expenditures and so we are not able to comment on actual dietary intakes, although substantial detail was collected on different food groups. It should also be recognized that increases in expenditures on ASFs may reflect in part, purchases of foods in these categories that are higher priced due to greater quality or more convenience, and not just increases in quantities of ASFs consumed [24], [37]–[39]. The nutritional impact of increase in ASF consumption expenditures is not possible to predict without knowing the food quantity in grams. This study focused on the household as a unit of ASF consumption, and the economic influences on ASF availability. This study did not address individual access: intra-household distribution may vary due to influences such as maternal knowledge [40], and cultural norms or labor and marriage market incentives that may discourage women and children from consuming ASFs [41]–[47]. While data on prepared foods were collected, they were not included in food expenditures as they could not be allocated to a food expenditure category. However, prepared food expenditures are included in the measure of total consumption expenditures.

Policy makers should consider the growing evidence base that suggests that efforts to increase income may be effective than food price regulations in achieving nutritional goals such as increasing demand for more nutrient-dense foods, and enhancing macro- and micronutrient consumption across a wide variety of nutrients [17], [32].

While the purpose of this study was not to evaluate the impact of various efforts to increase income, microcredit, cash transfers and employment-generating interventions are three promising approaches that can play an important part in increasing income, thereby improving nutrition [32], [48]. However, evidence demonstrating the impact of these efforts, along with nutritional education, is mixed. Regardless of the policies and programs governments and NGOs implement, identifying the most cost-effective means to improving consumption of ASFs requires detailed monitoring and evaluation as well as rigorous research, all of which has been too limited to date.

## Supporting Information

Figure S1 **Mean Expenditures on ASF subgroups as a Percent of ASF Expenditures by 2002 Total Consumption Quintiles in (a) 2002, (b) 2006 and (c) 2009.** In 2002 the percent of ASF expenditures on poultry, eggs and meat varied significantly (p<0.05) across 2002 total expenditure quintiles. For meat, the lowest quintile was significantly different from all others; the first quintile was different from the 2^nd^, and the third and fourth quintiles were different from the highest. For eggs, the lowest and second quintiles were both significantly different from all other quintiles. For poultry, the lowest quintile was different from the highest, the second quintile was different from the fourth and highest, and the third quintile was different from the highest. For dairy, the second and fourth quintiles were different and the fourth quintile was different from the highest. In 2006 percent of ASF expenditures on fish and eggs varied significantly by 2002 total expenditure quintiles. For fish, the lowest quintile was significantly different from the middle quintile and the second and third quintiles were significantly different. For eggs, the lowest quintile was significantly different from the fourth and highest quintiles, and the second and third quintiles were significantly different from the highest quintile. In 2009 the only significant differences between quintiles were in eggs (lowest different from fourth and highest quintiles) and poultry (lowest different from highest quintile).
(TIF)

Appendix S1 **Model Specifications.**
(DOCX)
