# Supplementary figures and images for: Households across All Income Quintiles, Especially the Poorest, Increased Animal Source Food Expenditures Substantially during Recent Peruvian Economic Growth
Source: PLoS One. 2014 Nov 5;9(11):e110961. doi: 10.1371/journal.pone.0110961 (PMC4220962; doi:10.1371/journal.pone.0110961)

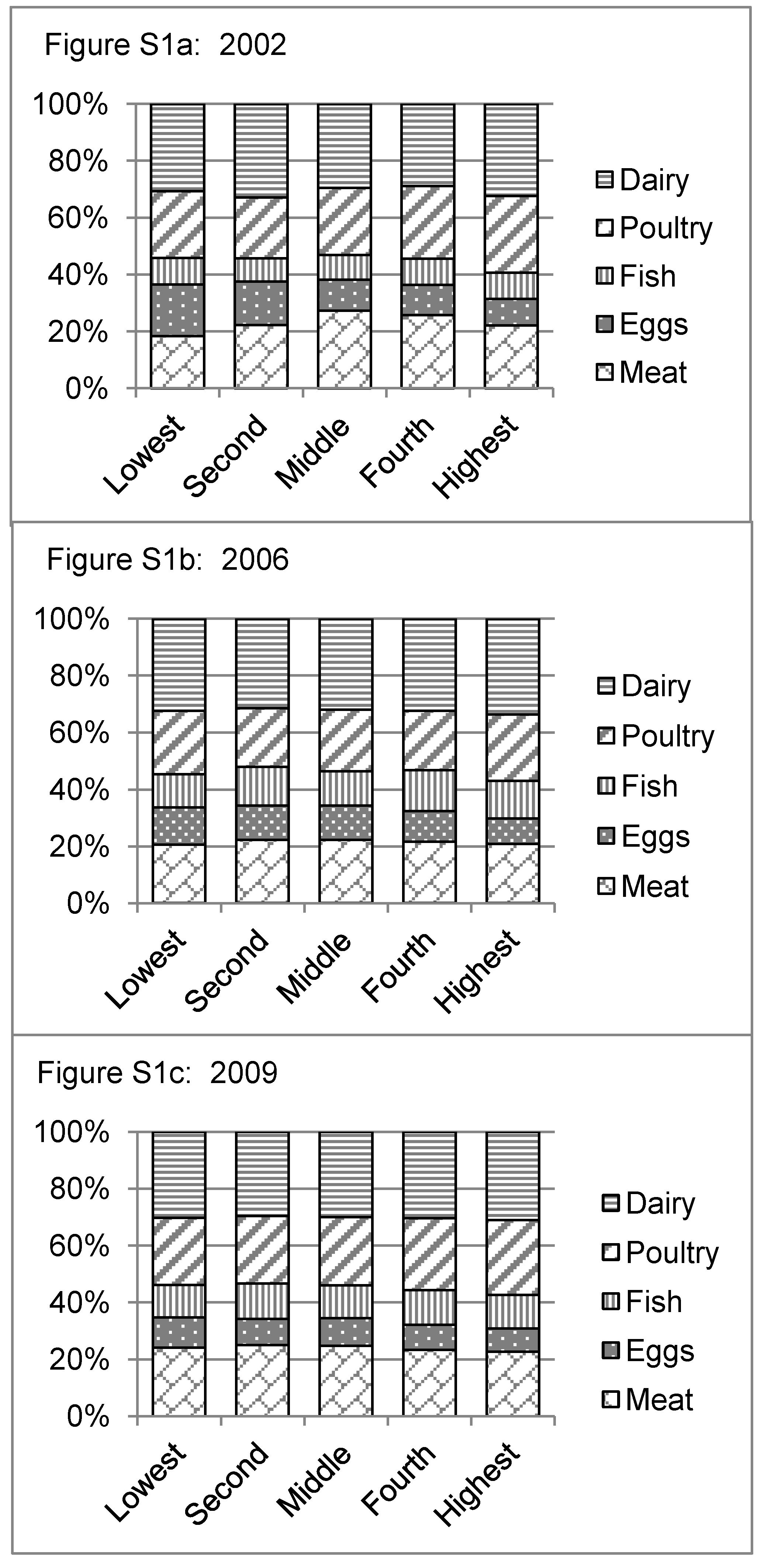

Supplement: Figure S1 — Mean Expenditures on ASF subgroups as a Percent of ASF Expenditures by 2002 Total Consumption Quintiles in (a) 2002, (b) 2006 and (c) 2009. In 2002 the percent of ASF expenditures on poultry, eggs and meat varied significantly (p<0.05) across 2002 total expenditure quintiles. For meat, the lowest quintile was significantly different from all others; the first quintile was different from the 2nd, and the third and fourth quintiles were different from the highest. For eggs, the lowest and second quintiles were both significantly different from all other quintiles. For poultry, the lowest quintile was different from the highest, the second quintile was different from the fourth and highest, and the third quintile was different from the highest. For dairy, the second and fourth quintiles were different and the fourth quintile was different from the highest. In 2006 percent of ASF expenditures on fish and eggs varied significantly by 2002 total expenditure quintiles. For fish, the lowest quintile was significantly different from the middle quintile and the second and third quintiles were significantly different. For eggs, the lowest quintile was significantly different from the fourth and highest quintiles, and the second and third quintiles were significantly different from the highest quintile. In 2009 the only significant differences between quintiles were in eggs (lowest different from fourth and highest quintiles) and poultry (lowest different from highest quintile). (TIF) [file pone.0110961.s001.tif]
