# Supplementary material for: Households across All Income Quintiles, Especially the Poorest, Increased Animal Source Food Expenditures Substantially during Recent Peruvian Economic Growth
Source: PLoS One. 2014 Nov 5;9(11):e110961. doi: 10.1371/journal.pone.0110961 (PMC4220962; doi:10.1371/journal.pone.0110961)
Supplement: Appendix S1 — Model Specifications. (DOCX) [file pone.0110961.s002.docx]

**Appendix S1:**

**Model specifications**

We began with simple ln relations for time period *t* and estimated the sequence of two relations pertaining to the solid arrows between the center boxes in Figure 1 discussed above:

$$\begin{matrix} ASF_{t}=a_{10}+a_{11}Food_{t}+a_{12}(Food_{t})^{2}+a_{13}Controls_{t}^{0}+a_{14}Controls_{t}^{u}+e_{1t}(1) \\ Food_{t}=a_{20}+a_{21}Consumption_{t}+a_{22}(Consumption_{t})^{2}+a_{23}Controls_{t}^{0}+a_{24}Controls_{t}^{u}+e_{2t}(2) \end{matrix}$$

Where *ASF* is the ln of ASF expenditures, *Food* is the ln of food expenditures, *Consumption* is the ln of consumption expenditures, *Controls* are controls that are observed (superscript *o*) and unobserved (superscript *u*), and the subscripts *t* for each variable refers to the *t^th^*period; *e* is a random stochastic term for each relation (first subscript) for period *t* (second subscript); and the *a*’s are coefficients for each relation (first subscript) for each variable in the relations (second subscript). The elasticity of ASF expenditure with respect to total food expenditures is *a_11_+ 2a_12_Food* and the elasticity of total food expenditures with respect to total consumption *a_21_+ 2a_22_Consumption*. Note that if there are no nonlinearities in the elasticities (i.e., *a_12_= a_22_=0*), the respective elasticities are given by the first terms (*a_11_, a_21_*) and are constant as *Food*  and *Consumption*  change – but if *a_12_* or *a_22_* is nonzero, the elasticity varies with *Food or Consumption* and thereby for poorer versus better-off households.

While estimates of relations (1) and (2) are of interest to better understand the sequence through which total consumption expenditures may affect ASF expenditures through food expenditures, it also is of interest to know the direct elasticity of ASF expenditures with respect to total consumption expenditures, which can be obtained from estimating:

$$\mathrm{ASF}_{t}=a_{30}+a_{31}Consumption_{t}+a_{32}(Consumption_{t})^{2}+a_{33}Controls_{t}^{0}+a_{34}Controls_{t}^{u}+e_{3t}(3)$$

For each relation [(1)-(3)] we undertook the following estimates:

Random Effects Model 1: The simplest specification with only the first two right-side variables using the two time periods together with random effects allowing for correlation of the disturbance terms across the two time periods under the assumption that the disturbance terms are not correlated with the right-side variables. Combining the two data rounds for these estimates increases the precision of the estimates. These models provided estimates of the three relevant sets of elasticities from relations (1)-(3) under the assumption that the absence of controls does not confound (bias) the estimates.

Random Effects with Controls Model 2: Model 1 plus standard observed controls (parental schooling, community wealth). These provided insight into whether the absence of observed controls biased the simple estimates and altered the estimated elasticities with such controls, all under the assumption that the absence of controls for the unobserved variables did not confound (bias) the estimates.

Fixed Effects Model 3: Model 3 with household fixed effects to control for unobserved fixed controls (e.g., some households may have preferences that lead to higher material wealth and consumption and to more ASF, so failure to control for such preferences biases upwards the consumption coefficient estimates), common unobserved trends shared experienced by all households (that, if not controlled and if correlated with the right-side expenditure variables also result may cause biases in the elasticities of the expenditure variables of interest) and for observed fixed controls (such as head and spouse education) to see if these estimates were preferred to the random effects estimates without control for the unobserved fixed effects and common unobserved trends and to see whether the elasticities of interest were affected by controlling for the unobserved fixed effects and common trends. We reported Hausman’s p that tests whether the fixed effects models are preferred over the random effects models. Based on the estimates of the multivariate relations, we estimated elasticities of ASF expenditures under multiple scenarios.
